# Modular Neuronal Assemblies Embodied in a Closed-Loop Environment: Toward Future Integration of Brains and Machines

**DOI:** 10.3389/fncir.2012.00099

**Published:** 2012-12-12

**Authors:** Jacopo Tessadori, Marta Bisio, Sergio Martinoia, Michela Chiappalone

**Affiliations:** ^1^Department of Neuroscience and Brain Technologies, Istituto Italiano di TecnologiaGenova, Italy; ^2^Department of Informatics, Bioengineering, Robotics and System Engineering, University of GenovaGenova, Italy

**Keywords:** bi-directional, *in vitro*, hippocampal cultures, confinement, micro electrode array, robot

## Abstract

Behaviors, from simple to most complex, require a two-way interaction with the environment and the contribution of different brain areas depending on the orchestrated activation of neuronal assemblies. In this work we present a new hybrid neuro-robotic architecture based on a neural controller bi-directionally connected to a virtual robot implementing a Braitenberg vehicle aimed at avoiding obstacles. The robot is characterized by proximity sensors and wheels, allowing it to navigate into a circular arena with obstacles of different sizes. As neural controller, we used hippocampal cultures dissociated from embryonic rats and kept alive over Micro Electrode Arrays (MEAs) for 3–8 weeks. The developed software architecture guarantees a bi-directional exchange of information between the natural and the artificial part by means of simple linear coding/decoding schemes. We used two different kinds of experimental preparation: “random” and “modular” populations. In the second case, the confinement was assured by a polydimethylsiloxane (PDMS) mask placed over the surface of the MEA device, thus defining two populations interconnected via specific microchannels. The main results of our study are: (i) neuronal cultures can be successfully interfaced to an artificial agent; (ii) modular networks show a different dynamics with respect to random culture, both in terms of spontaneous and evoked electrophysiological patterns; (iii) the robot performs better if a reinforcement learning paradigm (i.e., a tetanic stimulation delivered to the network following each collision) is activated, regardless of the modularity of the culture; (iv) the robot controlled by the modular network further enhances its capabilities in avoiding obstacles during the short-term plasticity trial. The developed paradigm offers a new framework for studying, in simplified model systems, neuro-artificial bi-directional interfaces for the development of new strategies for brain-machine interaction.

## Introduction

Algorithms based on classical models of computation cannot compare with living beings capabilities in terms of dealing with unexpected situations. Different fields of study, such as developmental biology (West-Eberhard, 2003; Gilbert, 2009), embodied cognition (Clark, 1997), evolutionary robotics (Bongard, 2011), seem to indicate as a likely cause for this shortcoming the lack of a developmental phase in traditional silicon-based technology. This process is especially evident in the Central Nervous System (CNS), where morphological changes, both reversible and permanent, occur on a wide range of different time scales. One possible way to deal with this issue is the realization of hybrid systems, where biological components could be exploited for their plastic properties.

In the recent past, several different hybrid model systems have been developed (DeMarse et al., 2001; Martinoia et al., 2004; Mussa-Ivaldi et al., 2010; Warwick et al., 2010; Kudoh et al., 2011), consisting of living neurons coupled to a robotic system. This solution allows the use of an artificial body whose dynamics can be easily and completely modeled, as opposed to the case of even the simplest animals. Furthermore, the exchange of information in a hybrid system can be limited to the desired level of complexity.

Following this “embodied neurophysiology” approach, we built a closed-loop electrophysiological system by interfacing a virtual mobile robot with a population of neurons, extracted from rat embryos and cultured over Micro Electrode Arrays (MEA; Novellino et al., 2007). The proposed paradigm represents an innovative, simplified, and controllable closed-loop system where it is possible to investigate the dynamic and adaptive properties of a neural population interacting with an external environment by means of an artificial body (i.e., the mobile robot). The main innovations of this experimental setup are: (i) the flexible software architecture at the base of the closed-loop experiments, here described in detail; (ii) the introduction of a modular network design. Starting from the observation of the high degree of modularity in the brain, different studies point out how such a property is likely to have a profound impact on neural activity (Hubel et al., 1977; Sporns et al., 2000; Derdikman et al., 2003; Kumar et al., 2010; Pan et al., 2010; Boucsein et al., 2011). In this work, we took advantage of the modular structure of the network to obtain a better separation between interacting cell assemblies. A significant improvement to previous works would be the added capability of inducing plastic changes in a controlled fashion. A step in this direction is taken in this setup by the use of a tetanic stimulation to enhance interconnected pathways to improve robot behavior (Jimbo et al., 1999; Chiappalone et al., 2008), following a collision with an obstacle. It is worth pointing out that the final objective of this work is not to achieve the best possible control of the robot: excluding any biological component would, at this stage, easily provide better performance and more reliable results. What is being developed here is groundwork for the integration of electronic systems and neural networks, with the twofold long-term objectives of taking advantage of neural plasticity in more complex control systems and performing closed-loops experiment to gage the computational and learning properties of relatively simple neural preparations.

## Materials and Methods

**Figure 1:**
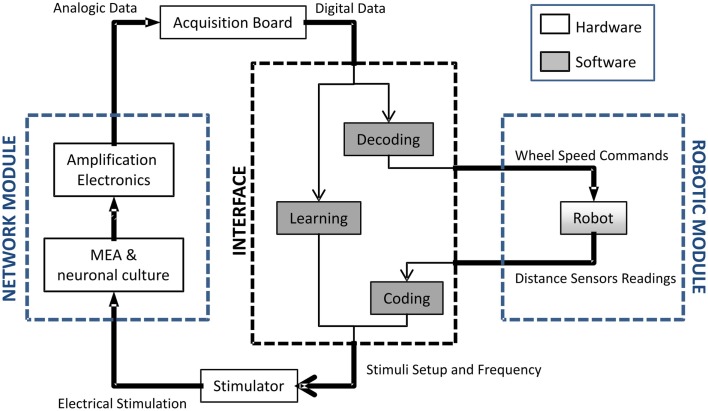
**Block diagram of the neuro-robotic architecture**. From left to right: (i) the network module, constituted by a network of living neurons coupled to a micro electrode array; (ii) a computer which hosts the developed software tool (i.e., HyBrain2) which manages the communication between the biological and the artificial part; (iii) the robotic module composed by a robot, either real or virtual, with sensors and actuators navigating into a circular arena with obstacles.

The setup developed for experiments of embodied electrophysiology is characterized by several different software, hardware and wetware components (Figure 1). The wetware part consists of hippocampal neurons cultured onto a standard 60-electrode MEA. The front-end electronics are constituted by a MEA1060-Inv-BC amplification system (Multichannel Systems, MCS, Reutlingen, Germany) and the computer used is a desktop machine (Dell Precision T5500, 2.66 GHz, 3.43 GB RAM) equipped with a DAQ E NI6255 (National Instruments, Austin, TX, USA) data acquisition board. An *ad hoc* adaptor was realized to interface the DAQ board with the amplification system. The software used for the management and acquisition is HyBrain2, a specifically developed software based on what is described in a previous work (Mulas et al., 2010): it allows control of all the parameters of the neuro-robotics experiments and performs the required data processing, such as the implementation of the coding, decoding and short-term plasticity schemes. Information is sent to the culture as a series of electrical stimulations through a Stimulus Generator 4002 (Multichannel Systems). Three different robots can be used for the experiments: two physical ones (Khepera II and its successor Khepera III, from K-Team, Zi les Plains-Praz, Switzerland) and a virtual implementation within the HyBrain2 architecture. The relevant elements of the robot are a set of distance sensors and two independently controlled wheels. Both the physical and the virtual ones have a circular arena with obstacles to move in. In all of the experiments, the task the robot is trying to perform is obstacle avoidance. While both physical robots have been tested and are properly working within the setup, in the following, only experiments with the virtual one are reported. The main problems with the physical robot are the fact that it requires actual tracking from an image to compute its position (which is both machine-time consuming and occasionally fails) and the non-idealities of its sensors: among the other, ambient lighting conditions have an impact on the performance of the infrared distance sensor and it has been reputed unwise to add such a factor of unpredictability at this stage of the development.

### Network module

#### Neuronal preparation: random and modular cultures

Dissociated neuronal cultures were prepared from hippocampi of 18-day-old embryonic rats (pregnant female rats were obtained from Charles River Laboratories). Culture preparation was performed as previously described (Frega et al., 2012). Briefly, the hippocampi of 4–5 embryos were dissected out from the brain and dissociated first by enzymatic digestion in trypsin solution 0.125% (30 min at 37°C) and subsequently by mechanical dissociation with a fine-tipped Pasteur pipette. The resulting tissue was re-suspended in Neurobasal medium supplemented with 2% B-27, 1% Glutamax-I, 1% Pen-Strep solution, and 10% Fetal Bovine Serum (Invitrogen, Carlsbad, CA, USA), at the final concentration of 60 k cells/ml.

**Figure 2:**
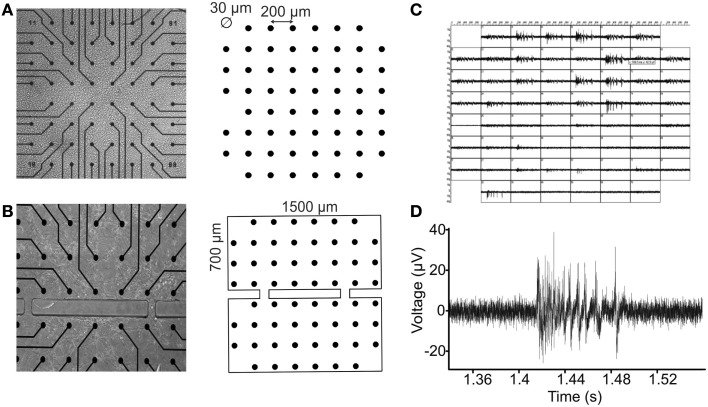
**Random and modular neuronal assemblies over micro electrode arrays**. **(A)** On the left, a random culture grown on a standard MEA device. On the right, the MEA layout is shown: a squared matrix of 59 micro electrodes (the missing one is the reference electrode), in which the inter-electrode distance is 200 μm and the micro electrode diameter is 30 μm. **(B)** On the left, a confined culture on a MEA substrate. On the right, the bi-compartmental system realized in PDMS with two interconnection microchannels. Compartments height is 700 μm, and width is 1500 μm. Microchannels height is 100 μm, and width is 50 μm. **(C)** Spontaneous electrophysiological activity of a confined culture of hippocampal neurons, registered from all the micro electrodes. **(D)** A typical hippocampal burst waveform recorded from a single channel.

Cells were afterward plated onto standard 60-channel MEAs previously coated with poly-d-lysine and laminin to promote cell adhesion (final density around 1200 cells/mm^2^) and maintained with 1 ml of nutrient medium (Figures 2A,B). They were then placed in a humidified incubator having an atmosphere of 5% CO_2_ and 95% air at 37°C. Half of the medium was changed weekly. Recordings were performed on cultures between 20 and 60 days *in vitro* (DIVs).

Considering the multitude of connections that usually forms in a random culture, a way to better control the network complexity consists of imposing a constraint to the neuronal cells growth along specific pathways (Chang et al., 2001; Boehler et al., 2012). To do this, a dual-compartment chamber with two interconnecting microchannels has been realized in polydimethylsiloxane (PDMS), a biocompatible, inert, and non-toxic polymer often used to this extent (Raichman and Ben-Jacob, 2008; Levy et al., 2012). The realization of the modular structures has been realized by replica molding using specific master with a previously developed technique (Berdondini et al., 2006). The obtained structures have been then placed on MEA substrates, in order to confine the growth of the neuronal cells that will be plated on it, as shown in Figure 2B.

#### Micro electrode arrays

Micro electrode arrays (Multichannel Systems, MCS, Reutlingen, Germany) consist of 60 TiN/SiN planar round electrodes (30 μm diameter; 200 μm center-to-center inter-electrode distance, see Figure 2A) arranged in an 8 × 8 square grid excluding corners. In some devices, one recording electrode is replaced by a larger ground electrode. Each electrode provides information on the activity of the neural network in its immediate area. A microwire connects each micro electrode of the MEA to a different channel of a dedicated amplifying system with a gain of 1100. The amplified 60-channel data is then conveyed to the data acquisition card which samples them at 10 kHz per channel and converts them into digital, 12 bit data (Figures 2C,D).

### Hybrain2 software

**Figure 3:**
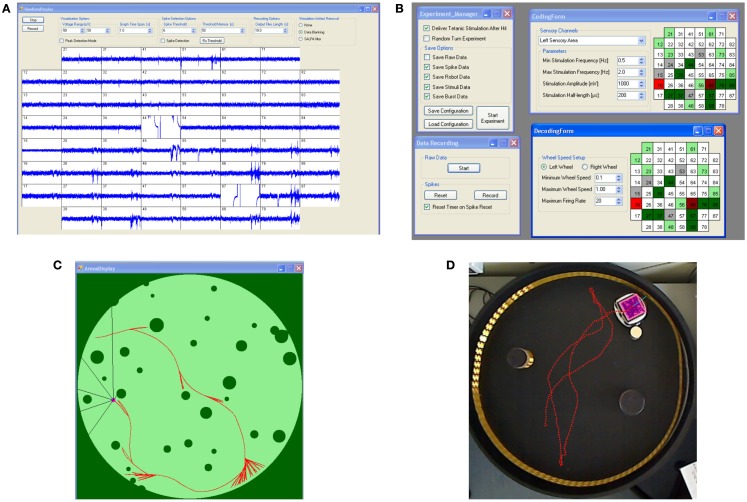
**Hybrain2 panels and robotic module**. **(A)** Raw electrodes data display panel, including options for data visualization, artifact filtering, and spike detection. **(B)** Several panels allow the configuration of coding and decoding algorithms and saving of data during experiments. **(C)** The robot arena panel shows the environment of the arena. In the case of a virtual robot, this can also be used to draw the arena itself. **(D)** The physical robot inside the arena where two obstacles are placed. The dotted red line represents the trajectory of the robot inside the arena.

The need for real-time access to data led to the adoption of a general-purpose acquisition card (NI6255, National Instruments, Austin, TX, USA) and required the development of a specific software: Hybrain2. The core of the program handles incoming data from the acquisition card and graphically displays them in a panel such as the one shown in Figure 3A. Spike detection options can be selected from this panel, such as threshold amplitudes or update times, as well as software blanking of stimulus artifacts. While a rather sophisticated algorithm (i.e., SALPA filtering; Wagenaar and Potter, 2002) for blanking has been included and validated, it has not been used in the described experiments, as it tends to compete for CPU-time with the rest of the system, leading to occasional resource starvation. In its current version, Hybrain2 does not make use of raw data other than for displaying. Instead, incoming data is processed by a spike detection algorithm (Maccione et al., 2009) whose output is a series of time stamps.

As explained later in more detail, both the coding and decoding algorithms for the closed-loop control of the robot are rate-based, therefore spike time stamps are a lossless representation of incoming data. Figure 3B shows the panels used for configuration of the parameters of these algorithms, such as selection of recording and stimulation electrodes, pulses amplitudes and lengths, and maximum and minimum allowed wheel speeds for the robot.

A module of the software is dedicated to managing the robot itself: in Figure 3C, a sample experiment with a virtual robot is shown. Here, the software is generating the robot environment as well as controlling all the relevant parameters of the robot itself, while, in the case of a physical robot (such as that in Figure 3D), the software provides a simple tracking feature on images provided by a webcam positioned over the arena and the required communication with the robot itself. All the data produced during experiments, including electrode readings, time stamps, and robot navigation data can be stored for later analysis both in text and/or binary format, while common parameters configurations can be saved and loaded in order to minimize experiment setup times and human errors.

### Robotic module

The robot, either virtual or physical, is basically a two-wheeled sensor platform: six infrared sensors are mounted on the robot at different angles, providing information about the distance of surrounding objects in different directions, whereas the speed profile of each wheel determine the direction and velocity of the robot itself.

The arena consists of an enclosed space containing several different round obstacles in random positions and the robot. A typical experiment with the virtual robot is shown in Figure 3C: the robot is moving in a 400 × 400 pixels circular arena, where dark green pixels represent obstacles or arena walls, whereas light green pixels are free for the robot to move in. The robot (small pink circle) is collecting information about its environment through its six sensors: each black line departing from the robot represents the line of sight of a different sensor; their angles are fixed with respect to the robot heading (in this case, 30°, 45°, and 90° on both sides of the robot direction), while the length of each line is equal to the distance from the robot center to the closest obstacle in the sensor direction. This distance defines the reading of the sensor: the output is 0 if the robot is in direct contact with an obstacle, 1 if the closest obstacle is at the maximum distance possible (the diameter of the arena, in this case). The three sensor readings on each side are averaged to provide the neuronal network with a single value per side.

In the case shown in Figure 3C, the robot is performing an obstacle avoidance task, as can be inferred by the red trajectory. The speed of a wheel is inversely proportional to the average of the sensor readings on the same-side, therefore the robot turns away from close obstacles. The ideal behavior of the robot is that of a Braitenberg vehicle (Braitenberg, 1984) in the case of no loss of information and no significant delays between sensor data collection and motor command execution. Obtaining a behavior as close as possible to this one is the goal of the coding-decoding-short-term plasticity process implemented here.

During experiments, collisions with obstacles or walls are unavoidable: following such an event, the robot moves back to a previous position in its path, at a fixed distance from any obstacle.

### Interfacing the network and the robotic module

#### Decoding scheme

Although many different decoding schemes are possible, so far the only one implemented has been a frequency rate-based algorithm (Adrian, 1928; Rieke et al., 1997; Martinoia et al., 2004). For this scheme, only a feature of the recorded signals is useful: the frequency of spikes at each location. A group of electrodes (i.e., a sub-population of neurons) on the MEA is selected and defined as the “output area” through the procedure described in the Section “Experimental protocol”. The number of spikes occurring over that area in 100 ms, non-overlapping windows constitutes the basis for calculating the motor signal for the corresponding wheel. In the current architecture, a linear relation is implemented between wheel speed and motor signal: if no spikes are detected in a time window, the corresponding wheel turns at a set minimum speed, increasing linearly with the number of detected spikes, up to a defined maximum rate. A low-pass filtering effect is added by taking into account previous samples, in order to smooth robot movements.

Dissociated neural networks are especially prone to bursting (Chiappalone et al., 2006) and this pattern of activity has been shown to code different information than just the sum of its spikes (Cozzi et al., 2006). A module for the detection of bursts has been already added to the Hybrain2 software, but its output is not yet part of the control loop of the robot.

For each wheel, the speed is therefore defined as:

$$\begin{array}{l} \omega_{i}=\{\begin{array}{cc} \frac{f_{i,t}+f_{i,t-1}}{2f_{i}^{\text{MAX}}}\left(\omega_{i}^{\text{MAX}}-\omega_{i}^{\min}\right)+\omega_{i}^{\min} & \text{for }f_{i}<f_{i}^{\text{MAX}}\\ \omega_{i}^{\text{MAX}} & \text{for }f_{i}\geq f_{i}^{\text{MAX}} \end{array} \end{array}$$

where subscript *i* denotes wheel side, ω is the wheel speed, and *f_i_,*t** is the averaged firing rate over all the electrodes corresponding to the *i*-th recording area at time sample *t*. ω^MAX^, ω^min^, and *f*^ MAX^ are parameters set by the experimenter before the start of the experiment.

#### Coding scheme

Likewise, the coding scheme is linear and rate-based: two groups of electrodes are defined as “input areas” and assigned to the sensors on the left and right side of the robot body. The details for area selection are fully explained in the Section “Experimental Protocol”. Each sensor provides a reading, normalized to 1 for an object in direct contact with the robot and 0 for an object at the far end of the designed arena (while this behavior is nearly ideal for the virtual robot, it is far from so in the case of the physical robot, as already mentioned in the Section “Materials and Methods”. The readings from the sensors on the same-side of the robot are then averaged and coded back to the corresponding sensory area. As mentioned before, the coding is linear and frequency based: a fixed stimulus is delivered at the sensory area at a frequency directly proportional to the averaged, same-side sensors readings. The stimulation rate for each input region is determined as:

$$\begin{array}{l} s_{i}=\left(s_{i}^{\text{MAX}}-s_{i}^{\min}\right)r_{i}+s_{i}^{\min} \end{array}$$

where *s_i_* is the stimulation rate of the *i*-th input area and *r_i_* the normalized average of all the sensor readings on the corresponding side of the robots, whereas $s_{i}^{\text{MAX}}\;s_{i}^{\min}$ are user-set parameters fixing the maximum and minimum stimulation rate.

#### Short-term plasticity protocol

In order to progress toward the desired behavior, it is necessary to define a learning rule that allows a modification of connectivity between input and output areas by rewarding “good behavior,” while discouraging “bad behavior.” The effect of tetanic stimulation in these networks was already demonstrated by our group and by others in the past, showing that a 20 Hz stimulation should strengthen the synaptic connections of receiving neurons (Jimbo et al., 1999; Tateno and Jimbo, 1999; Madhavan et al., 2007; Chiappalone et al., 2008; le Feber et al., 2010). In all these papers the effect of the tetanus on the change of firing rate was studied in a time frame comparable to that of our experiments (30 min to 1 h). Additionally, in a previous paper from our group (Chiappalone et al., 2008), we were able to demonstrate that a single tetanic shock to a neuronal network had an immediate effect in terms of increase in the Post Stimulus Time Histograms (PSTH) area (i.e., increase in the number of spikes evoked by a stimulus), a medium-term effect (i.e., few hours after the tetanus delivery), and a long-term effect (i.e., 1 day after the tetanus delivery).

The above observations have been used to define the learning rule in the current implementation of the software: following each robot collision, a 2-s-long, 20 Hz stimulation is delivered to the same-side input area. The rationale for this choice is that collisions are usually caused by poor correlation between stimulation in an input area and detected activity in the corresponding output area, thus making the network responses to stimulation insufficient to steer the robot in the correct direction. Our hypothesis is that tetanic stimulation strengthens all participating connections, thus correcting the problem, as demonstrated in the studies cited above. A tetanic stimulation induces short-term plasticity effects which allow the groups of neurons involved in the obstacle avoidance tasks to fire at a higher frequency, thus inducing the corresponding wheel to increase the angular velocity. Since input-output regions were selected according to connection strength (see Experimental Protocol below), this should increase responses detected from the desired electrodes upon delivery of a stimulus from the input electrodes. This bring to a generalized strengthening of connections in the network and to an improvement in the driving of the robot.

### On-line processing of electrophysiological signals

#### Spike detection

The electrophysiological signals acquired from MEA electrodes must be preprocessed in order to remove the stimulus artifact and to isolate spikes from noise. The spike detection algorithm uses a differential peak-to-peak threshold to follow the variability of the signal and a set of controls are performed in order to make the algorithm as reliable as possible (Maccione et al., 2009). The threshold is proportional to noise SD and is calculated separately for each individual channel (typically as six or seven times SD) before the beginning of the actual experiment (i.e., during phase 1 of the protocol described below).

#### Blanking of stimulus artifact

Stimulus artifacts are detected when the recorded signal exceeds a defined threshold much higher than the one used for spike detection. The artifact is then suppressed by canceling the first samples in the spike train occurring immediately after it, corresponding to a signal blanking of 4 ms after stimulus delivery.

### Experimental protocol

The typical experimental protocol followed in this work consists of a five-step procedure:

1. Monitoring of the spontaneous activity of the culture;
2. Test stimulus from a set of electrodes in order to choose the I/O of the network, necessary for the connection with the robot;
3. 20-min run without short-term plasticity protocol
4. 20-min run with short-term plasticity protocol
5. Evaluation of the robot’s performances on the basis of specific navigation’s parameters.

During the first step of the experimental session, spontaneous activity of the network is subject to observation, in order to determine, empirically, which electrodes are the most likely candidate as “input” sites (i.e., sites from which stimulation must be delivered). Typical features to look for in this phase are a sustained mean firing rate (i.e., sufficient number of spikes per second, usually higher than 0.1 spikes/s) and patterns of activity not synchronous with other regions. The best candidates (usually a set of 8–10 sites) are then selected for the second step of the experiment. From each of the candidate “input” channel, in turn, a 500-μs, 1.5 V peak-to-peak, bipolar square wave is delivered every 5 s, until a total of 40 stimuli per channels have been delivered, while spiking activity is detected from other electrodes.

At the end of this phase, for every stimulation electrode involved, 59 PSTH are generated (Chiappalone et al., 2007): these graphs report the average number of spikes detected from each electrode in the 600 ms following each stimulation and therefore provide information on the strength of the connections in the culture. Through a custom-made script developed in the Matlab environment (The Matworks, Natick, MA, USA), the generated PSTHs are then compared in order to look for areas that present a significant degree of specificity, i.e., where responses are not elicited by stimulation delivered from all the electrodes, but from some of them. In this way, it is possible to define an output (recording) area that will respond mostly to stimulation from the corresponding input area, while remaining silent during stimulation from the opposite input area (cf., see “Input and Output Sites of a Neuronal Population” of the Results).

During steps 3 and 4, the robot is left free to roam the arena with the rules described above, with a tetanic stimulus following each collision with an obstacle delivered during step 4. If the starting hypotheses hold true, this will progressively drive the network toward the desired condition of reliable and specific evoked responses.

Finally, we collect the data on the robot performances. In order to verify the neural-based behavior of the robot, we compared the results obtained (i) in a neuron-controlled experiment (a MEA with living neurons grown on, bi-directionally connected to the robot), (ii) in a open-loop experiment (a MEA with living neurons grown on, but without sensory feedback), and (iii) in an “empty” MEA experiment (a MEA with culturing medium only). In case (ii), the robot performs in a way imposed by the spontaneous firing rate of the neural network, usually in a random pattern, while in the case of the “empty” MEA (iii) the robot basically drives in a straight line (see the Supplementary Videos and Closed-Loop Robot Navigation of the Results).

### Database of experiments, data analysis, and statistics

Experiments on a total of *N* = 17 different cultures, ranging from 20 to 60 DIV, have been conducted: 11 of those were random hippocampal cultures, while the other six experiments were conducted on hippocampal cultures, divided into sub-populations by a confinement mask, as described above. Those six cultures were also compared for spontaneous activity evaluation with a subset of six random cultures (age range of the subset: 21–42 DIV).

In order to highlight differences in term of synchronization between the two populations, a cross-correlation algorithm was applied to spike trains, a technique already introduced previously (Frega et al., 2012). Briefly, the cross-correlation function (i.e., cross-correlogram) is defined by the incidence of a spike at electrode *y* after that a spike was fired at electrode *x*. More specifically, given two spike trains (i.e., *x* and *y*) from two electrodes of a MEA, we count the number of spikes in the *y* train within a time frame around the spikes of the *x* train of ±T (in the order of tens of milliseconds), using bins of amplitude Δτ (usually set at multiple of the sampling frequency). The correct *C_xy_*(τ) is obtained by means of a normalization procedure, by dividing each element of the array by the square root of the product between the number of peaks in the *x* and the *y* train. If the obtained *C_xy_*(τ) shows a distribution that clearly deviated from flat, electrodes x and y are considered correlated. For each cross-correlogram *C_xy_*(τ) we then estimated the coefficient *C*_peak._ *C*_peak_ represents the value of the cross-correlogram in an area around the maximum detected peak and it is usually evaluated in order to quantify the *correlation level* among two recording channels. The statistical distribution of all *C*_peak_ values was computed for the two experimental groups during spontaneous activity (i.e., random vs. modular cultures). For each robot run, two different parameters have been computed in order to evaluate the performance of the robot, namely the average distance traveled by the robot between hits (measured in pixels) and the average number of hits per second. The virtual robot is implemented so that following a collision against an obstacle, it is immediately moved to the last location where its center was at least 20 pixels away from any other object. Since the robot radius is 5 pixels, the lower limit for the average distance traveled by the robot during each robot run is that of 15 pixels.

**Figure 4:**
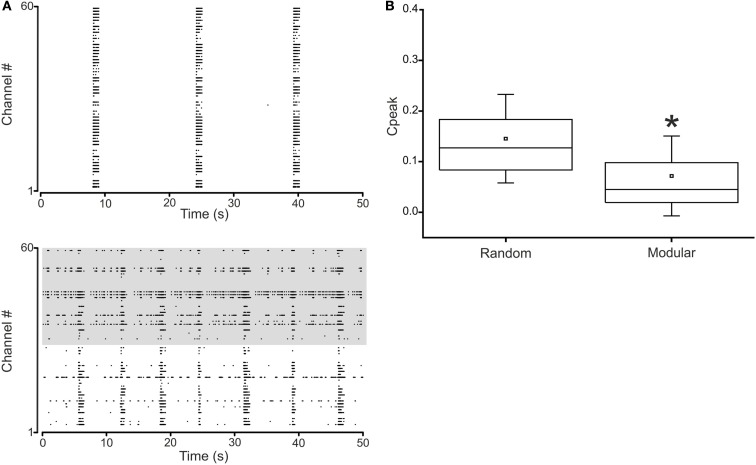
**Spontaneous activity in random and modular hippocampal networks**. **(A)** Top. Raster plot of the activity exhibited by a random hippocampal culture (50 s of activity acquired from a representative culture of 28 DIV). Bottom. Raster plot of the activity exhibited by a modular hippocampal culture (50 s of activity acquired from a representative culture of 25 DIV). The activity of 59 electrodes is depicted: each small vertical bar represents a spike, each line an electrode. **(B)** Box-plot of the cross-correlation peaks in *N* = 6 random and *N* = 6 modular cultures. Box range: percentile 25–75; box whiskers: percentile 5–95; line: median; square: mean. Mann–Whitney test for not-normal data, significance level = *p < 0.05.

**Figure 5:**
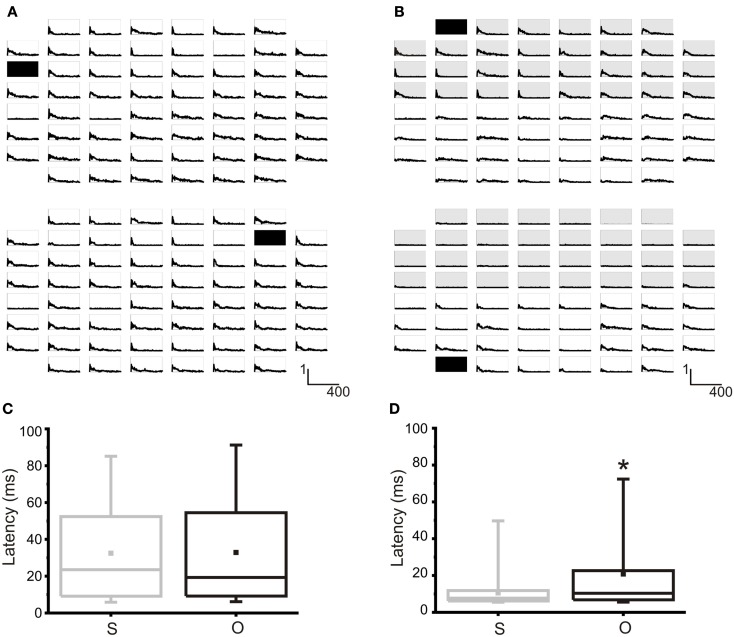
**Evoked activity in random and modular hippocampal networks**. **(A)** *Top*. PSTH map obtained from 59 channels as a consequence of the stimulation from electrode 13 (black square). *Bottom*. PSTHs obtained by stimulating electrode 72 (black square) in the same network. *X*-axis: time (0, 400) ms, bin 4 ms; *Y*-axis: probability of evoking a spike. **(B)** *Top*. PSTH map obtained from 59 channels as a consequence of the stimulation from electrode 21 (black square) in the top compartment of a confined network. *Bottom*. PSTHs obtained by stimulating electrode 28 (black square) in the bottom compartment of the same confined network. Shaded area indicates the top compartment. *X*-axis: time (0, 400) ms, bin 4 ms; *Y*-axis: probability of evoking a spike. **(C)** Box-plot of the latency from the first evoked spikes in the same (S) or other (O) compartment with respect to stimulating electrodes. No statistical differences can be noted in a random culture. *N* = 11 random cultures. **(D)** Box-plot of the latency from the first evoked spikes in the same (S) or other (O) compartment with respect to stimulating electrodes. In a modular network, the latency between the stimulus and the first evoked spike is statistically lower for the electrodes belonging to the same cluster of the stimulating electrodes. *N* = 6 modular cultures. Box range: percentile 25–75; Box whiskers: percentile 5–95; line: median; square: mean. Mann–Whitney test for not-normal data, significance level = *p < 0.05.

**Figure 6:**
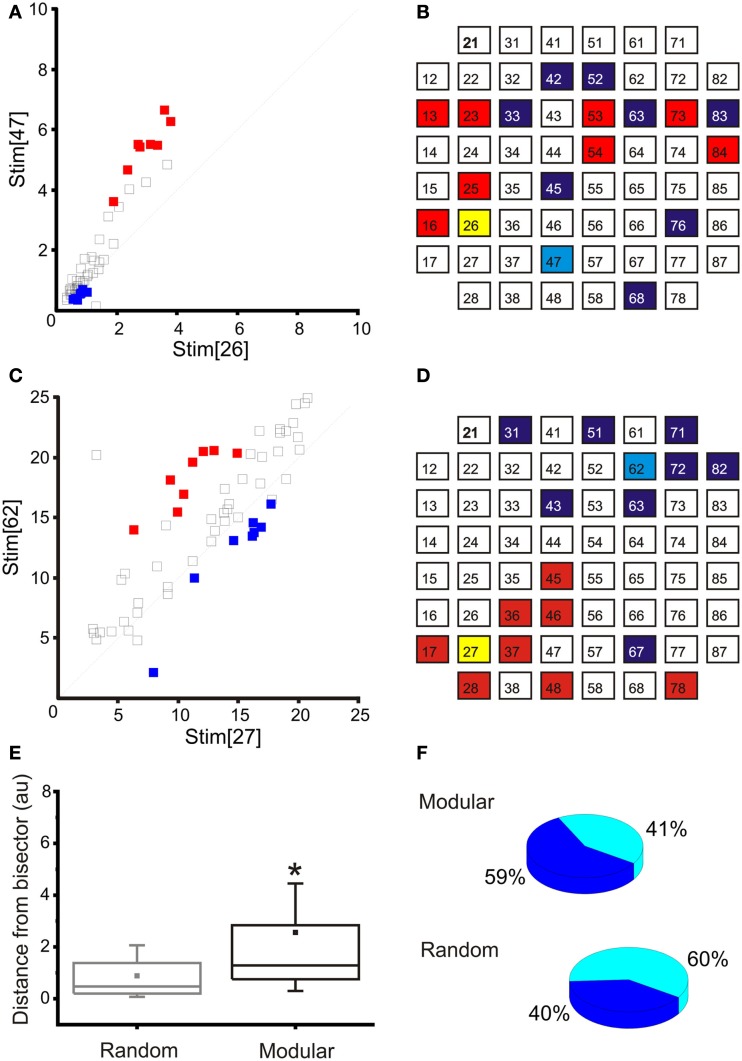
**Input-output selection**. **(A)** Map obtained in a representative “random” culture for the selection of the output sites, given two inputs sites (e.g., 26 and 47): red, left recording area; blue, right recording area. **(B)** Schematic representation of the input (yellow and light blue) and respective recording (red and blue) areas for the same experiment reported in A (“random” culture): note that the selected electrodes are quite spread over the entire recording area. **(C)** Map obtained in a representative “confined” culture for the selection of the output sites, given two inputs sites (e.g., 27 and 62): red, left recording area; blue, right recording area. **(D)** Schematic representation of the input (yellow and light blue) and respective recording (red and blue) areas for the same experiment reported in **(B)** (“confined” culture): note that the selected recording electrodes are close to the stimulating electrode and they follow the structure of the underlying network. **(E)** A box-plot representing the distances from bisector of the selected recording electrodes in the set of random and confined cultures used within this study (*N* = 11 random and *N* = 6 modular cultures). The distribution of the distances in the modular case is significantly higher than in the random case. Box range: percentile 25–75; box whiskers: percentile 5–95; line: median; square: mean. Mann–Whitney test for not-normal data, significance level = *p < 0.05. **(F)** Pie chart representing the percentage of networks in which at least 50% of the recording electrodes were selected in the same compartment of the stimulating electrode. The percentage is higher for the modular networks (*N* = 11 random and *N* = 6 modular cultures).

**Figure 7:**
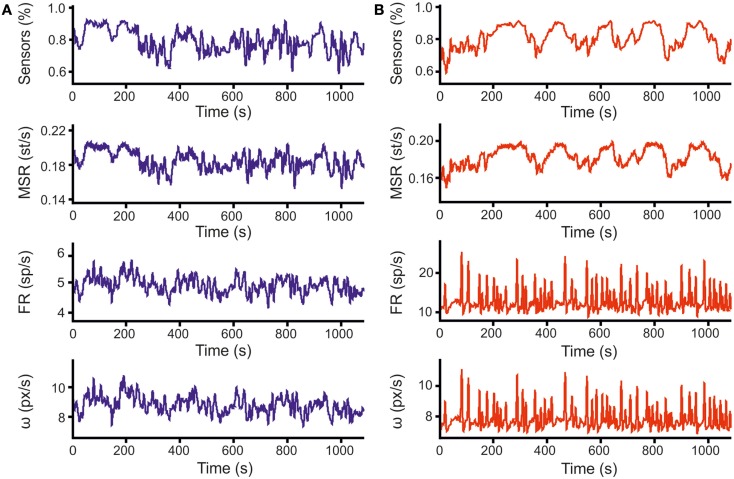
**Closed-loop data samples**. **(A)** The three graphs represent 1100 s of data recorded during a robot run under close-loop control. From top to bottom, the graphs represent: (i) the average readings of the three proximity sensors on the left side of the robot, ranging from 1 (obstacle in contact with the robot) to 0 (obstacle at the distance of the arena diameter); (ii) mean rates of delivery of stimulation (i.e., Mean Stimulation Rate, MSR); (iii) mean firing rates by averaging over all the electrodes belonging to the same recording area (i.e., Firing Rate, FR); (iv) speed of the robot wheels, expressed in pixels per second, as computed according to Eq. 1 from firing rates. Data for stimulation and firing rate are point events at times of delivery (for stimulations) or detection (for spikes), while sensor data and wheel speeds are sampled at 10 Hz. The graphs reported above are obtained after low-pass filtering of actual data (sliding Gaussian window over 100 samples – 10s, with an alpha value of 2.5). **(B)** Same set of graphs as **(A)**, displaying information for sensors and wheel of the right side.

**Figure 8:**
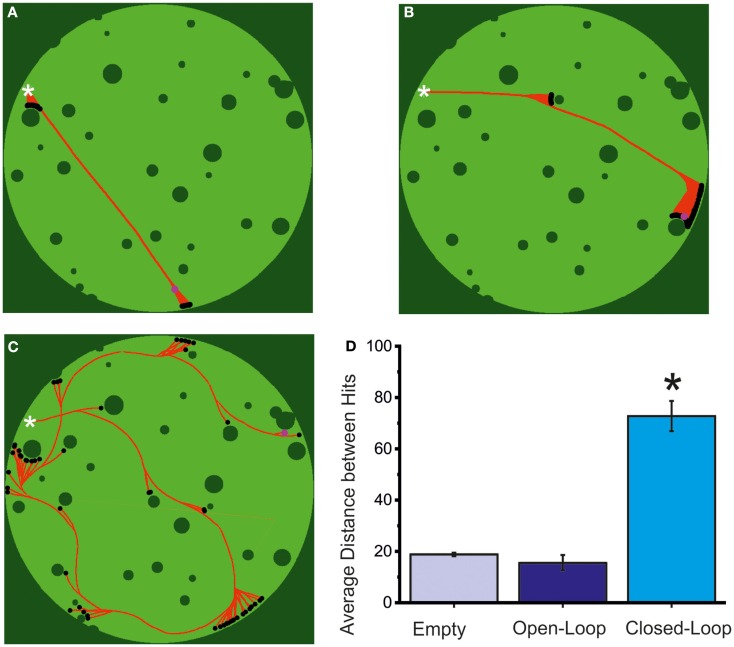
**Robot navigation and evaluation of the closed-loop system**. **(A)** Reconstruction of a 20-min long robot trajectory, in an empty MEA configuration. The white cross marks the starting position of the robot and the red path its movement during the observation period, up to its final position (pink circle, in the upper right corner). Dark green pixels are either arena walls or obstacles, while light ones are free for the robot movement. Black dots represent robot impacts with the environment. Total lack of biological material on the MEA prevents a closing of the sensory-motor loop. As a consequence, the robot shows a total inability to navigate its environment. The small changes in robot heading are likely false positives in the spike detection algorithm on background noise or stimulation artifacts. As can be inferred from the image, though, their total impact is almost null and the robot moves almost precisely in a straight line. **(B)** Reconstruction of a 20-min long robot trajectory in open-loop. During this robot run the control loop has been opened by stopping stimulation to the neural culture. As a result, the robot is, similarly to the previous case, lacking any capability of navigating its environment. Changes in robot direction are, in this case, provoked by the spontaneous activity of the neural network. **(C)** Reconstruction of a 20-min long robot trajectory in closed-loop. While the amount of obstacles hit by the robot shows that control is not perfect, the robot is able to take advantage of sensory information to extricate itself from all the situations encountered in a limited amount of time and hits. **(D)** Performance of the neuro-controlled robot during an obstacle avoidance task in terms of the mean distance between two consecutive collisions, calculated in pixels. The values are obtained in *N* = 5 experiments for the empty and the open-loop case and in the *N* = 17 experiments reported in the text (light blue = empty MEA; blue = open-loop MEA; cyan = closed-loop MEA). The closed-loop experiments give the best results. Statistical analysis was carried out by using one-way ANOVA (*p < 0.05) for normal distributions (Kolmogorov–Smirnov test of normality), while for mean comparison both the Tukey and the Bonferroni tests were used.

Statistical tests were employed to assess the significant difference among diverse experimental conditions. The normal distribution of experimental data was assessed using the Kolmogorov-Smirnov normality test. According to the distribution of the data, we performed either parametric (e.g., ANOVA, Figure 7) or non-parametric (e.g., Mann–Whitney U test, Figures 4–6 and 8) tests and *p* values < 0.05 were considered significant. Statistical analysis was carried out by using OriginPro (OriginLab Corporation, Northampton, MA, USA).

## Results

### Network dynamics: Spontaneous activity in random and modular networks

Hippocampal cultures grown *in vitro* over MEAs show a spontaneous (i.e., ongoing) activity, similar to that exhibited by *in vivo* systems during their development (Ben-Ari, 2001) or during deep sleep (Corner, 2008). Their electrophysiological behavior is characterized by spontaneous spiking which becomes synchronized with the maturation of the network, giving rise to phenomena called “bursts,” network bursts (Pasquale et al., 2010) or network spikes (Eytan and Marom, 2006). These network bursts are the fingerprints of a steady-state in which the network dynamic found a balance between excitation and inhibition (on average 70–80% of neurons are excitatory ones and the remaining 20–30% is constituted by inhibitory interneurons). Such state can be easily pharmacologically disrupted by acting on the glutamatergic as well as on the gabaergic receptors or by adding neuromodulators (Keefer et al., 2001; Eytan et al., 2004; Frega et al., 2012). Another possibility to alter such stereotyped behavior is to introduce modularity (i.e., interconnected populations) instead of having a single uniform and random culture (Raichman and Ben-Jacob, 2008; Shein Idelson et al., 2010; Kanagasabapathi et al., 2012).

Figure 4 shows the spontaneous activity from a representative random (Figure 4A, top) and a modular culture (Figure 4A, bottom) during the fourth week of development. While in the random culture the activity is highly synchronized and packed in the form of “network bursts” (van Pelt et al., 2004; Pasquale et al., 2010), in the modular culture we can identify two different temporal patterns of activity with moments of synchronized bursts interleaved with sparse spiking periods. Synchronized network bursts spread to the whole culture also in the modular networks, even if, globally, modular cultures are much less correlated than the random ones (Figure 4B).

### Network dynamics: Evoked activity in random and modular networks

It is possible to electrically modulate the activity of the network by means of electrical stimulation. The typical response of a network can be evaluated through the Post Stimulus Time Histogram (PSTH, cf., see Materials and Methods). In Figure 5A the maps of the PSTH obtained as a consequence of the stimulation from site 13 (top) and site 72 (bottom) are reported in a non-confined culture. Typically, the PSTH is characterized by an “early response,” lasting 20–40 ms, and by a late response, lasting more than 100–200 ms, usually due to the generation of an evoked burst synchronized over the whole network (Gal et al., 2010). The integral calculated over the PSTH profile represents the average number of evoked spikes at a specific site and it is used for quantifying the strength of the connection between a specific stimulation site and all the recording ones (Chiappalone et al., 2008). This parameter is at the base of the choice of the input-output connections for our neuro-robotic studies (cf., see Input and Output Sites of a Neuronal Population). Figure 5B reports the maps of the PSTH obtained in a modular network. When stimulation is delivered from site 21 (top compartment, Figure 5B top), mainly the electrodes of the top compartment respond to the stimulation. Few activations can be observed also in the bottom compartment, but with a dominant late response and an almost absent early one. In the same network, when stimulation comes from one electrode of the bottom compartment (electrode 28, Figure 5B bottom) practically only that compartment responds to the stimulus.

To further test the actual confinement of the evoked responses, we also analyzed the distribution of the mean latencies (i.e., the distance between the stimulus and the first evoked spike) obtained for each couple of stimulation-recording electrodes (Mainen and Sejnowski, 1995; Tateno and Jimbo, 1999): simply by eye, it is clear that the evoked response is (mostly) limited to the compartment hosting the stimulation electrode. Figures 5C,D reports the distribution of the latencies from the electrodes in the same compartment (i.e., top or bottom) of the stimulating electrode (S) compared to those from the electrodes in the other compartment (O). Only in the case of confined networks (Figure 5D) the two distributions are statistically different, being the latencies evaluated in the electrodes belonging to the same compartment of the stimulation significantly lower than those of the electrodes in the other compartment. This proves that dividing the neural network in two sub-populations has indeed an effect on stimulus response.

### Input and output sites of a neuronal population

The simplest architecture that can be adopted for the proposed task includes two electrodes to deliver coded sensory information, one for each set of sensors. While the same could be said for output sites, the point of interest in this work was the response of the network, therefore a set of 8–10 electrodes is chosen to act as output sites for each wheel.

The main disadvantage in dealing with dissociated cultures instead of experimental models with a preserved neural structure is the lack of predefined architecture. For this reason, before starting an experiment, a procedure has been performed to define the stimulation (sensory input) and recording (motor output) areas of the network. During this procedure (i.e., phase 2 of our experimental protocol, cf., see Materials and Methods), we stimulated the cultures by delivering trains of 40 electrical stimuli (1.5 V peak-to-peak, biphasic pulses, 500 μs total duration) from 8 to 10 sites in a serial way. Then, the PSTH area (i.e., the number of spikes in the 600 ms following each stimulation) between each pair of stimulation-recording electrodes is computed and the related maps, like the one reported in Figures 6A,C, are produced. The coordinates of each square in that map represent the PSTH areas at a specific recording site relative to stimulation from the two stimulating sites reported on the axis (Stim[26] and Stim[47] in Figure 6A, for example). All the possible input-output combinations are explored and only the pathways producing “selective” responses are retained. These “selective” pathways are identified by pool of recording sites with respect to a couple of stimulating sites for which the responses measured fall far away from the bisector (i.e., pool of recording site closer to the axis).

Those specific pathways of sensory-motor activations can be then conveniently utilized for driving the robot and for implementing simple reactive behaviors (e.g., obstacle avoidance). Figures 6B,D report the selected inputs (i.e., two electrodes, one for the left and one for the right area) and output regions, characterized by eight electrodes each, corresponding with maps Figures 6B,D, respectively for two representative cultures (i.e., random and modular).

The presence of a confinement structure tends to generate networks showing a higher degree of functional separation (i.e., selectivity), as well as a physical one, when compared to totally random networks: as can be seen in Figure 6E, the average distance from the bisector of the evoked response pair is significantly increased in the case of the modular network. The geometry of the stimulation-recording pairs is also affected, as they are more likely to be clustered together on the same half of the culture (Figure 6F).

### Closed-loop robot navigation

All the parameters relevant to the movement of the robot are recorded during the experiment. In Figures 7A,B, more than 1000 s of signal recordings are plotted (Figure 7A for the left side and Figure 7B for the right side). The top panels are showing sensory information, with the blue trace representing the average value of proximity sensors on the left side of the robot and the red one the average value of those on the right. In the second graph, a measure of stimulation is shown, expressed as the mean stimulation rate. The third line of graphs reports the firing rates, measured in spikes per second; wheel speeds (shown in the lower graph, expressed in pixels per seconds), closely follow neural activity.

The results of the behavior described so far can be observed in Figures 8A–C, where a virtual arena is shown along with the path drawn by the robot (in red) in a 20-min long robot run, respectively in an “empty” experiment (Figure 8A), an open-loop experiment (Figure 8B) and finally a closed-loop experiment (Figure 8C). While collisions are fairly frequent even in the latter case the behavior of the robot is still much closer to the desired one rather than in an open-loop configuration, or (obviously) in the absence of a biological substrate. As can be observed from the graph in Figure 8D, the average path traveled between hits is significantly higher in the case of a close-loop.

### Impact of modularity and tetanic stimulation on robot navigation

**Figure 9:**
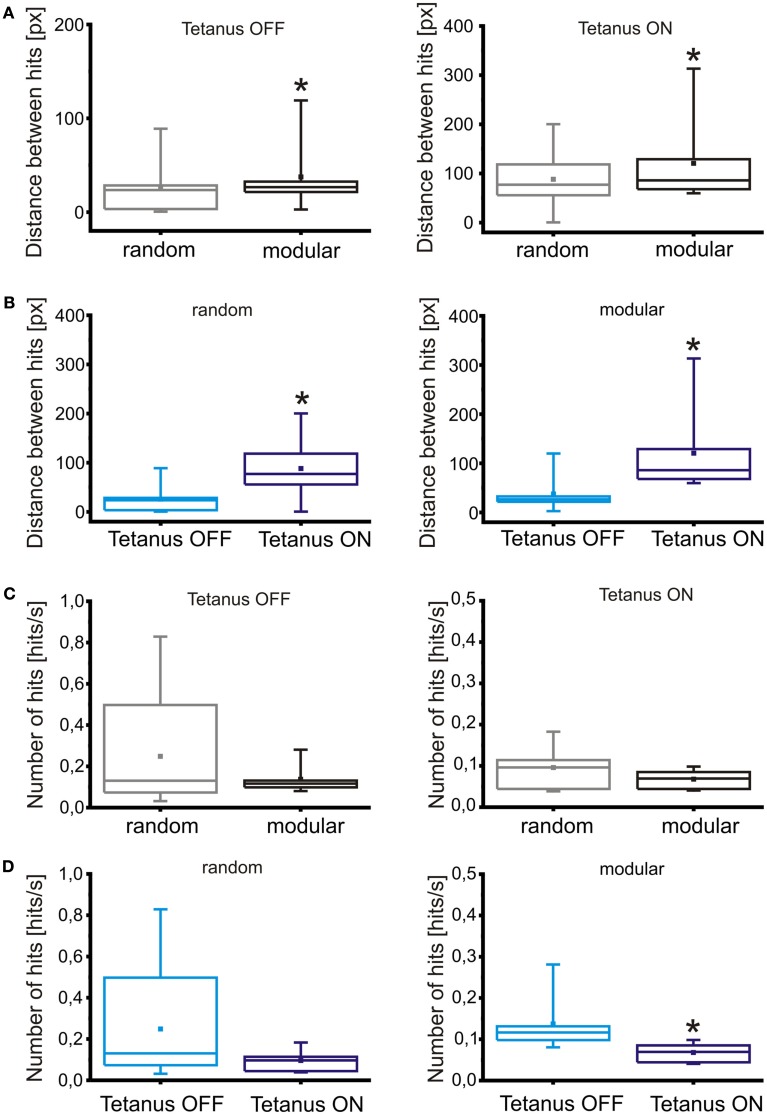
**Impact of modularity and tetanic stimulation on robot navigation**. **(A)** Comparison of robot performances in random and modular networks, in the absence or presence of tetanic stimulation (respectively, left and right graph), evaluated as average distance (in pixels) between consecutive hits. **(B)** Comparison of robot performances between different conditions of tetanic stimulation, in random (left graph) and modular networks (right graph), evaluated as average distance between hits. **(C)** Comparison between robot performance in random and modular networks, in the absence or presence of tetanic stimulation (respectively, left and right graph), evaluated in terms of average number of hits per second. **(D)** Comparison of robot performances in different conditions of tetanic stimulation, in random (left graph) and modular networks (right graph), evaluated in hits per second. All the values are obtained in the experiments described in text (*N* = 11 experiments for the random condition, *N* = 6 for the modular), with a tetanic stimulation session following each standard robot run. Box range: percentile 25–75; box whiskers: percentile 5–95; line: median; square: mean. Statistical analysis was carried out by using Mann–Whitney test for not-normal data, significance level = *p < 0.05.

**Figure A1:**
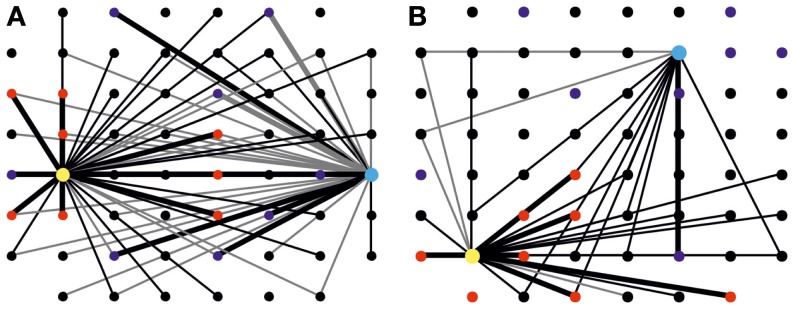
**Maps of changes in effective connectivity**. **(A)** Changes in effective connectivity occurring during a tetanic stimulation experiment. The large dots, in yellow and light blue, represent electrodes used for delivery of both tetanic stimulation and sensory information for the left and right inputs. The smaller dots in blue and red indicate the position of electrodes used for recording from the two “motor” areas. Change in effective connectivity is defined as the difference in the area of PSTHs measured after and before the short-term plasticity experiment, divided by the average of these two values. Variations greater than 20% are represented as lines on the maps, with gray and black lines indicating, respectively, a decrease and an increase in functional connectivity. Only connections involving either stimulating electrode have been represented for clarity, with thicker lines highlighting the connections used in the closed-loop control of the robot (left input-left output and right input-right output areas). This map, in particular, is displaying the change in connectivity observed on a random culture during a 30-min short-term plasticity experiment. In this culture, tetanic stimulation led to a widespread increase of connection strengths involving the electrode represented in yellow, while those involving the one in light blue underwent a mixed change, with about half of them resulting strengthened and half of them weakened. **(B)** Same map as **(A)** obtained from recordings on a modular culture before and after a 30-min short-term plasticity experiment. While tetanic stimulation was delivered to both the yellow and light blue electrodes (respectively in the “lower” and “upper” halves of the culture), only one of the sub-populations was significantly affected, with a diffuse increase in connectivity.

Despite the improvement in performance of the closed-loop scenario compared to the control cases, robot collisions against obstacles are still a frequent occurrence in random networks. Observation of PSTHs reveals that random networks show a very high degree of connectivity, with evoked responses showing a strong overlap regardless of the stimulating electrodes position (Figure 5A). The introduction of a confinement mask shows a marked separation in the responses obtained from stimulation, as can be observed from Figure 5B. This, in turn, leads to a reduction in the amount of “cross talk” between input and output channels, with a consequent increase in the navigation performance of the robot. Figures 9A,C compare the improvement in performance between the random network structure and the modular one. Specifically, Figure 9A shows the comparison between performances evaluated as the average distance between consecutive collisions in different conditions (without and with tetanic stimulation, respectively on the left and right graphs), while Figure 9C displays the same performances evaluated through a different parameter, average number of hits per second. The tetanic stimulation leads to a further improvement in the performance, especially when performed on a network with a modular geometry, as can be observed in Figures 9B,D: the first couple of graphs show the increase in performance following the introduction of the tetanic stimulation routine (in a random network, left, and in a modular one, right) evaluated as distance between collisions, while the graphs in Figure 9D show the performance obtained in the same experiment as average number of hits per second. Examples of changes in effective connectivity obtained in modular and random networks can be observed in Figure A1 in the Appendix. Even if quantification will be necessary, preliminary analyses of changes in connectivity show that tetanic stimulation does affect the network response, by strengthening the connections on one side and weakening or not affecting the connections on the opposite side.

While all of the described comparisons yield statistically significant results in the case of the average distance parameter, it is not the case for the average number of collisions: the only condition that causes a large enough change to be significant is the introduction of a tetanic stimulation on a modular network.

## Discussion and Future Development

In this paper we successfully interfaced, in a bi-directional way, a network of neurons coming from the hippocampus of embryonic rats with a virtual robot. The robot, which has sensors and wheels, is forced to move in a static arena with obstacles and its task consists in avoiding collisions. Looking at the spontaneous electrophysiological activity of the network, we first select a set of possible “inputs,” then we evaluate the evoked response of the entire culture by delivering patterns of electrical stimulation. This procedure allows us to select the “outputs” of our network. Then, by applying a linear rate-based decoding strategy, we were able to transform the spike frequency into velocity and the sensory information collected by the robot “eyes” into stimulation frequency for our neurons. The behavior of the robot during the closed-loop experiments resulted significantly better than that in open-loop (i.e., without any sensory feedback) or the “empty” MEA condition, proving that the activity driving the robot is actually neural-based (cf. Figure 8). In general, these results prove that an *in vitro* network of biological neurons can control an external agent. While ours is not the first setup to achieve this goal, in our knowledge, no previous work reports an extensive set of experiments like the ones we performed (DeMarse et al., 2001; Martinoia et al., 2004; Novellino et al., 2007; Bakkum et al., 2008; Kudoh et al., 2011), but, rather they focus on a single thesis supported by data obtained from a limited number of analogous preparations. Here, we introduce for the first time statistical comparisons obtained on a sizable number of different preparations with highly different spiking behaviors, such as those observed on random and modular networks. Furthermore, bi-modularity of cultures is introduced here for the first time in the context of closed-loop interfaces and its impact is shown to be relevant for the performance of the embodied agent.

Early experiments on random networks showed the tendency of these cultures to evolve toward a degenerate state where mostly network-wide synchronous activity can be observed. The addition of a confinement mask and the consequent modularity qualitatively changed the behavior of the network, preventing or at least strongly reducing the appearance of synchronized network bursts (cf. Figure 4). This change alone was enough to provide a significant increase in the performance of the robot (cf. Figures 5, 6, and 9). These results lead to two possible investigation lines on the same experimental setup: increasing the modularity of the network might allow more complex behavior to emerge, while chronic stimulation since the day of plating might be used in future experiments to define functionally but not physically distinct sub-populations of neurons within the same culture.

Another point of novelty in our approach has been the systematic use of tetanic stimulation on hippocampal cultures over MEA. Previous approaches aiming at demonstrating plasticity in neuronal assemblies by using stimulation protocols from embedded extracellular electrodes were always applied to cortical cultures (Jimbo et al., 1999; Madhavan et al., 2007; Chiappalone et al., 2008; Stegenga et al., 2010). Here we used hippocampal cells and we proved that tetanic stimulation worked successfully, providing an increase in performance both in random and modular networks (cf. Figure 9). A further analysis on data is being conducted to determine whether it is possible to define a clear relationship between spontaneous activity of the network and its impact on the observed changes in connectivity strength, since the patterns of induced change proved to be more complex than expected (see Figure A1 in the Appendix for a preliminary example of effective connection changes induced by tetanic stimulation). This could allow the design of a more successful learning scheme. The exact biological mechanisms linking performance increase and tetanic stimulation are still unclear and further investigations and targeted experiments are needed. Along this direction, the use of pharmacological manipulation could allow to change the state of the network and thus to investigate roles of synaptic transmission and receptors involved in the process of adaptation and learning depending on specific stimulation protocols.

As expected, the final performance of the robot is worse than what was possible to achieve without including biological components in the closed-loop (data not shown): for the task of obstacle avoidance, it would be possible to program the robot so that it can perform the navigation task with no risk of hitting obstacles. However, our neuro-robotic framework proved to be a valid tool for the study of mechanisms of neural coding and the computational and adaptive properties of neuronal assemblies with the final goal to facilitate progress in understanding neural pathologies, designing neural prosthetics, and creating fundamentally different types of artificial or hybrid intelligence.

## Conflict of Interest Statement

The authors declare that the research was conducted in the absence of any commercial or financial relationships that could be construed as a potential conflict of interest.

## Supplementary Material

The Supplementary Material for this article can be found online at http://www.frontiersin.org/Neural_Circuits/10.3389/fncir.2012.00099/abstract

Supplementary Video S1 **Video of a closed-loop robot run**. This video of a virtual robot run is running at 40× real speed. The arena is composed of dark green solid obstacles and light green “floor” which the robot can move upon. The magenta circle is the virtual robot itself, the red dots highlight the path followed by the robot center over time, while black circles represent hits against obstacles. While the amount of obstacles hit by the robot shows that control is not perfect, the robot is able to take advantage of sensory information to extricate itself from all the situations encountered in a limited amount of time and hits.

Supplementary Video S2 **Video of an “empty MEA” robot run**. This video of a virtual robot run is running at 40× real speed. The arena is composed of dark green solid obstacles and light green “floor” which the robot can move upon. The magenta circle is the virtual robot itself; the red dots highlight the path followed by the robot center over time, while black circles represent hits against obstacles. The starting direction of the robot in this trial is rotated 90° clockwise with respect to the other two shown videos. Total lack of biological material on the MEA prevents a closing of the sensory-motor loop. As a consequence, the robot shows a total inability to navigate its environment. The small changes in robot heading are likely false positives in the spike detection algorithm on background noise or stimulation artifacts. As can be inferred from the video, though, their total impact is almost null and the robot moves almost precisely in a straight line.

Supplementary Video S3 **Video of an open-loop robot run**. This video of a virtual robot run is running at 40× real speed. The arena is composed of dark green solid obstacles and light green “floor” which the robot can move upon. The magenta circle is the virtual robot itself, the red dots highlight the path followed by the robot center over time, while black circles represent hits against obstacles. During this robot run the control loop has been opened by stopping stimulation to the neural culture. As a result, the robot is, similarly to the previous case, lacking any capability of navigating its environment. Changes in robot direction are, in this case, provoked by the spontaneous activity of the neural network.
